# The Public and Professionals Reason Similarly about the Management of Non-Native Invasive Species: A Quantitative Investigation of the Relationship between Beliefs and Attitudes

**DOI:** 10.1371/journal.pone.0105495

**Published:** 2014-08-29

**Authors:** Anke Fischer, Sebastian Selge, René van der Wal, Brendon M. H. Larson

**Affiliations:** 1 Social, Economic and Geographical Sciences Group, James Hutton Institute, Aberdeen, Scotland, United Kingdom; 2 Aberdeen Centre for Environmental Sustainability (ACES) and School of Biological Sciences, University of Aberdeen, Aberdeen, Scotland, United Kingdom; 3 Department of Environment and Resource Studies, University of Waterloo, Waterloo, Ontario, Canada; University of Kent, United Kingdom

## Abstract

Despite continued critique of the idea of clear boundaries between scientific and lay knowledge, the ‘deficit-model’ of public understanding of ecological issues still seems prevalent in discourses of biodiversity management. Prominent invasion biologists, for example, still argue that citizens need to be educated so that they accept scientists’ views on the management of non-native invasive species. We conducted a questionnaire-based survey with members of the public and professionals in invasive species management (n = 732) in Canada and the UK to investigate commonalities and differences in their perceptions of species and, more importantly, how these perceptions were connected to attitudes towards species management. Both native and non-native mammal and tree species were included. Professionals tended to have more extreme views than the public, especially in relation to nativeness and abundance of a species. In both groups, species that were perceived to be more abundant, non-native, unattractive or harmful to nature and the economy were more likely to be regarded as in need of management. While perceptions of species and attitudes towards management thus often differed between public and professionals, these perceptions were linked to attitudes in very similar ways across the two groups. This suggests that ways of reasoning about invasive species employed by professionals and the public might be more compatible with each other than commonly thought. We recommend that managers and local people engage in open discussion about each other’s beliefs and attitudes *prior* to an invasive species control programme. This could ultimately reduce conflict over invasive species control.

## Introduction

There is a longstanding debate about the differences between lay and expert knowledge in the ecological realm [1], and about the challenges for biodiversity governance where expert and lay perspectives are incongruent with each other [2]. Several studies have addressed differences between lay and expert knowledges and perspectives in relation to nature conservation and the management of landscapes and biodiversity [3], [4]. For example, previous research has concluded that the general public perceives biodiversity inaccurately relative to the ‘actual’ biodiversity levels assessed by experts, and thus diagnosed a public lack of ecological knowledge [5]. According to a recent review of scientists’ views of the public [6], such conclusions may indicate a ‘deficit model’ of public understanding that is still prevalent among many scientists, who tend to see ‘the public’ as a homogenous, amorphous body that needs to be educated.

However, there is an increasingly strong critique of the deficit model. This critique originates in the sociology of science and argues that the boundary between laypeople and experts is diffuse and permeable [7]–[9]: “Expert knowledge is open to reappropriation by anyone with the necessary time and resources to become trained, and […] there is a continuous filtering back of expert theories, concepts and findings to the lay population” [10]. Recognition is growing, also in the ecological realm, that the dichotomy between experts and laypeople is less clear than it might have seemed twenty years ago [1], and several recent studies in conservation science provide evidence for this alternative view by highlighting the links between concepts used in both expert and lay domains [4], [11], [12]. Most empirical research in this context focuses on comparisons of these understandings, and reports that experts and laypeople differ in some aspects, for example, in their risk perceptions, preferences for landscapes and management scenarios, or knowledge of plant and animal species [3], [5], [13], [14], whereas they might concur in others, for example, in their appreciation of woodland features [15]. While such findings might be interesting, insights into structures of expert and lay thought [4] and the mechanisms behind such similarities and differences remain scarce.

In this study, we investigate the beliefs and attitudes of laypeople and experts regarding the perceived non-nativeness and invasiveness of animal and plant species. This is relevant to the debate over the deficit model because, even in recent publications, some invasion biologists still maintain a clear dichotomy between themselves and the public: “Although most invasion scientists endorse a normative commitment towards biodiversity, their proper role as scientists, in terms of public discourse, is to educate citizens in a way that informs debate within society about how to *think about* and manage invasions” [16] (emphasis added; see [17] for other examples). Such statements do not seem to acknowledge the permeability described above [10], the potential diversity of public views of invasive species, or the range of perspectives even among invasion biologists [18], [19]. One consequence attributed to the divide between laypeople and experts is that invasive species control has sometimes been delayed by public opposition, which has led to explicit calls for more insights into the obstacles to “public buy-in” to species management [20]–[23].

To date, a number of studies have investigated either public [24]–[29] or expert [18], [19], [30], [31] views of non-native invasive species. In some instances, such studies have focused on one species, for example, buffel grass (*Cenchrus ciliaris*) [23], and it remains unclear whether their results can be generalised. Only very few studies have examined views of experts and the public simultaneously. While the terminology is sometimes used ambiguously, the term ‘experts’ can include practitioners, academics and hobby experts in invasive species management, whereas ‘scientists’ are a subset of experts active in academic research on this topic. ‘Professionals’ can be defined as a subset of experts who have specific skills or knowledge related to invasive species and whose work engages directly with them [10]. A study in the Doñana region of Spain included both professionals and non-professionals, and found that some groups of the public, such as nature tourists, held similar knowledge and attitudes as conservation professionals [32]. Another study, in Scotland, identified the arguments that members of the public and scientists used to support their views on a range of management options for invasive species [33]. These arguments consisted of perceived species characteristics, such as the harmfulness of a species in relation to nature or the economy.

The present study builds on this literature and quantitatively contrasts how members of the public and professionals, from a large and cross-cultural sample, view the management of multiple species. Most critically, and unlike most previous studies (see [4] for an exception), we do not solely concentrate on comparisons of single constructs, for example, knowledge about species or attitudes towards management options, because such an approach necessarily remains at a somewhat descriptive level. Instead, we investigate the arguments for and against certain management options by explicitly investigating the statistical *relationships* between beliefs about species and attitudes towards their management. In the terminology used in science education, we thus distinguish between content knowledge and ways of reasoning [34], [35]. This allows us to compare the *ways* in which beliefs inform attitudes, rather than just either beliefs or attitudes on their own.

According to social psychological frameworks, such as the Theory of Planned Behaviour [36], beliefs are conceptualised as factors influencing attitudes, and ultimately, behaviour. We define beliefs here as the subjective probability that an object (here: an animal or plant species in a given context) has a certain attribute [36]; for example, that a person considers red deer (*Cervus elaphus*) to be rare in Scotland. Beliefs, whether held by laypeople or experts, can thus be considered as a subjective form of knowledge. Attitudes are evaluations of objects or behaviours with some degree of favour or disfavour [37]; for example, that red deer should be managed or not. A strong relationship between relevant beliefs and attitudes, for example, a link between the belief that “red deer cause economic damage in Scotland” and the attitude that “red deer should be controlled”, suggests that individuals’ views are well embedded in their cognitive contexts and thus not volatile and unstable [38].

Previous research on attitudes towards biodiversity management has identified several beliefs that are likely to be relevant to the management of invasive species, and that form a useful basis to study differences between professionals and the public [33], [39], [40]. Here, we examine the relationships between beliefs and attitudes in a quantitative manner, and hypothesise that species believed to be non-native will be seen in greater need of management than those perceived to be native. Moreover, we predict that species regarded as invasive – here operationalised as three separate concepts, namely (a) abundance, (b) harm caused to nature and (c) harm caused to the economy – will be seen to require greater management efforts [25], [33], [39], [41]. We thus explicitly distinguish between four different aspects (nativeness, abundance, harm caused to nature, and harm caused to the economy) of the complex of notions associated to non-nativeness and invasiveness. Finally, we hypothesise that there will be strong support for management of species that are regarded as unattractive [29], [40], [41] or easy to control [13].

In addition, these relationships between beliefs and attitudes towards management might vary between professionals and laypeople. For example, aesthetic attractiveness has been described as a factor that is considered especially by laypeople, rather than experts [42], whereas we would expect factors related to conservation science, such as nativeness and detrimental effects on nature, to constitute a stronger influence on professionals’ attitudes than public ones [33]. Here, we explicitly investigate the degree to which the strength and the nature of these relationships differ between professionals and members of the public.

## Methods

### Ethics statement

The project underwent an ethics review and received full clearance, specific to the study, from the Office of Research Ethics of the University of Waterloo (#16311). In Scotland, no additional ethics approval was obtained, as neither the James Hutton Institute nor the School of Biological Sciences at Aberdeen University had an ethics committee for social scientific research at the time (2010). However, although the ethics clearance from the University of Waterloo referred only to the Canadian participants, the survey instrument used in Scotland was the same as the one employed in Ontario. Ethical issues were thus minimised. The return of completed questionnaires was considered as inferred informed consent, given that anonymity and confidentiality were explicitly granted and questionnaires did not include any information that could be used to identify individual respondents. All data was thus anonymised prior to analysis.

### Survey administration and sampling

We conducted a questionnaire-based survey in two geographic areas, Scotland (UK) and Ontario (Canada), to increase the generality of our results (and obtain a sufficiently large sample size of professionals for quantitative analysis, see below). Both areas are English-speaking (a factor that facilitates cross-country application of questionnaires), and both have an industrialised Western cultural context, a landscape composed of both agricultural and semi-natural habitat, and broadly similar climates and thus functional types of biota (Köppen-Geiger classification for Scotland: Cfb; Ontario: Dfb, i.e., similar rainfall patterns (f) and warm summers (b), while temperature ranges largely overlap (C/D) [43]).

Rather than investigating differences in views between the two study areas, we aimed to compare beliefs, attitudes and, importantly, their relationships across the two sub-samples, i.e., the public and professionals (hereafter called ‘groups’). We focus here on professionals, who we define as a sub-group of experts, not necessarily with scientific backgrounds, whose work engages directly with invasive species [10]. This includes members of governmental, non-governmental and research organisations as well as company representatives. Note that our public sample, as it is randomly drawn, may include not only laypeople, but also the occasional professional.

Samples of the general public (Table 1) were designed to be representative of their target population, that is, the adult residents of Scotland and Ontario, respectively. In Scotland, we used a commercial dataset that built on the electoral roll and had been complemented by other data sources that also included non-voters. Addresses were randomly selected from the dataset. A printed copy of the questionnaire (Questionnaire S1) was then sent to 1,500 addresses in June 2010, together with a prepaid return-envelope and a cover letter that referred to species management in general and did not mention biological invasions. A reminder postcard was sent two weeks after the initial questionnaire. About 20% of the addressees returned completed questionnaires.

**Table 1:** Overview of sample sizes and demographic characteristics (gender, age) per sub-sample.

	Public		Professionals	
	*Scotland*	*Ontario*	*Scotland* *	*Ontario*
	(n = 276)	(n = 270)	(n = 93)	(n = 93)
***Gender*** (female)	58.3%	55.2%	32.3%	40.9%
***Age***				
18–30 years	12.8%	20.1%	15.2%	22.6%
31–60 years	53.1%	60.4%	75%	70.9%
>60 years	34.1%	19.5%	9.8%	6.5%

*Professionals in the ‘Scotland’ sample were based across the whole of Great Britain.

In Ontario, we used an online version of the questionnaire that was distributed to a survey panel administered by a market research company, GMI (Global Market Insite, Inc., http://www.gmi-mr.com). The respondents were sampled from southwestern Ontario telephone area codes across rural and urban areas, but excluding Toronto (a disproportionally large metropolitan centre compared to those in Scotland). The panel completed the survey in November and December 2010.

Professionals involved with invasive species were directly contacted through pertinent e-mail lists, namely the list of the Non-Native Species Secretariat for Great Britain (https://secure.fera.defra.gov.uk/nonnativespecies/home/index.cfm) and the Ontario Invasive Plant Council (http://www.ontarioinvasiveplants.ca). A cover e-mail and a link to an on-line version of the questionnaire were sent to all members of these mailing lists. For consistency with the public sample in Scotland, the professionals (who were distributed over the whole of Britain) were asked to specifically consider the species in question in a Scottish context. As the surveys were also advertised in two relevant newsletters, response rates are difficult to estimate but were a minimum of 59% and 16% for Scotland and Ontario, respectively. The overall sample included n = 732 respondents (n = 186 professionals; n = 546 public, Table 1).

### Questionnaire design

Our survey was structured by two sets of species (one for each country) that included a range of non-native, native, invasive and non-invasive species in order to obtain variation in the respective beliefs. Each set contained five species representing the following types: native mammal (two species), non-native mammal, native tree, and non-native tree/tall shrub. ‘Non-native’ refers here to species introduced from one geographical region into another one and is synonymous with ‘alien’ [44]. The term ‘invasion’, by contrast, denotes a species’ spread [45], [46], often with implicit or explicit connotations of harm caused by this spread [44]. The main criterion for species selection was familiarity: species had to be well known to a broad lay audience as widespread lack of familiarity would have resulted in meaningless data. We also aimed to identify species that were as similar as possible across the two study areas. From a bigger set of species explored in several rounds of pre-testing, we chose five species per study area for inclusion in the final version of the questionnaire (Table 2). Although the opossum, a rabbit-sized marsupial, has been found in eastern Canada for about 150 years, it has recently been spreading northward in response to climate change [47] and can thus be considered as non-native. We also included the beaver (*Castor fiber/canadensis*), a mammal that is found in both study areas and which is culturally significant; the beaver is native to Ontario, and reintroduced to Scotland after historical extirpation from the UK. All species were shown in a small photograph.

**Table 2:** Species used in the questionnaire, per study area.

Species type	Scotland	Ontario
Native mammal	Red deer (*Cervus elaphus*)	White-tailed deer (*Odocoileus virginianus*)
Non-native mammal	Grey squirrel (*Sciurus carolinensis*)	Virginia opossum* (*Didelphis virginiana*)
Native tree	Scots pine (*Pinus sylvestris*)	Eastern white pine (*Pinus strobus*)
Non-native tree/tall shrub	Rhododendron (*Rhododendron ponticum*)	Scots pine (*Pinus sylvestris*)
Significant mammal	Eurasian beaver* (*Castor fiber*)	American beaver (*Castor canadensis*)

*See text for discussion.

Draft versions of the questionnaire were jointly developed by the authors and extensively pilot-tested, both qualitatively and quantitatively. Belief and attitude statements had been identified in earlier qualitative work [33]. The final version included six items that captured *beliefs* about species, phrased as semantic differentials [48] that included the opposite ends of a spectrum, namely: (i) ugly – beautiful; (ii) beneficial – detrimental to the economy; (iii) beneficial – detrimental to nature; (iv) non-native – native; (v) uncontrollable – controllable; and (vi) rare – overabundant. Three additional pairs of attributes elicited *attitudes* towards species management: (i) not a severe problem – a severe problem; (ii) no need to reduce species numbers – need to reduce species numbers; and (iii) killing this species is not ok – killing this species is ok. For each belief and attitude item, following common practice in questionnaire surveys, five response options were offered (−2, −1, 0, 1, 2). The belief items also allowed ‘don’t know/not applicable’ answers.

### Data analysis

To obtain a robust measure of attitudes towards species management, we combined the three five-level attitude items (see above) into a single attitude index by a simple computation of the average score. Inter-item reliability as measured by Cronbach’s α across the whole sample and all five species was α = 0.801. Cronbach’s α captures the degree to which different items reflect the same construct and is thus a measure of internal consistency of a group of items that are intended to address the same idea. Although α tends to vary with the number of items (the more items, the higher α), values above 0.6 are commonly considered acceptable for studies on attitudinal concepts [49].

We ran linear mixed models in SPSS Version 21 including the data for all five species pairs, both study areas (Ontario and Scotland) and both groups (public and professionals), with the attitude index as a response variable. As fixed effects we entered the categorical variables ‘study area’ and ‘group’, and the interval-scaled variables ‘beauty’, ‘impact on economy’, ‘impact on nature’, ‘nativeness’, ‘controllability’ and ‘abundance’ (i.e., the six beliefs). To explicitly test whether professionals employ beliefs differently from members of the public we fitted all two-way interactions between the individual beliefs and ‘group’ (e.g., beauty×group).

In an exploratory step of the analysis, we also included all interactions between these beliefs and ‘study area’. However, only the term ‘study area×abundance’ was significant (F = 60.04, estimate = 0.19, p<0.001), which was not surprising given the large overall effect of abundance on the response variable (F = 834.3, estimate = 0.38, p<0.001). The direction of the effect suggested that the relationship between attitudes and perceptions of abundance were stronger in the Scottish sample than in the Canadian one. As no further significant impacts of study area could be detected and the investigation of country differences was not the focus of this study, we omitted these interaction terms from the analysis to concentrate on a comparison between the views of professionals and the public.

Because of our specific interest in the significance levels of the interactions between the beliefs and ‘group’, we used Type III calculation of the sums of squares. The order of the explanatory variables entering the model was thus based on the strength of the partial correlation with the dependent variable, starting with the strongest. The procedure gives – unlike Type I – an equal chance for main effects and interactions to be entered first in the model and is thus more likely to detect interactions present in the data. The factors ‘species’ and ‘individual respondent’ were entered as random effects to reflect the study design and thus structure of the data (i.e., each respondent expressed their beliefs and attitude towards the management of five different species). Parameters were computed using maximum likelihood estimation. Visual inspection of residual plots did not reveal any obvious deviations from assumptions of normality and homoscedasticity. Models that combined both the public and professional samples (with interactions fitted) yielded the same results as a model with a split sample; we therefore report only the combined model.

## Results

### Beliefs about species and attitudes towards management

**Figure 1:**
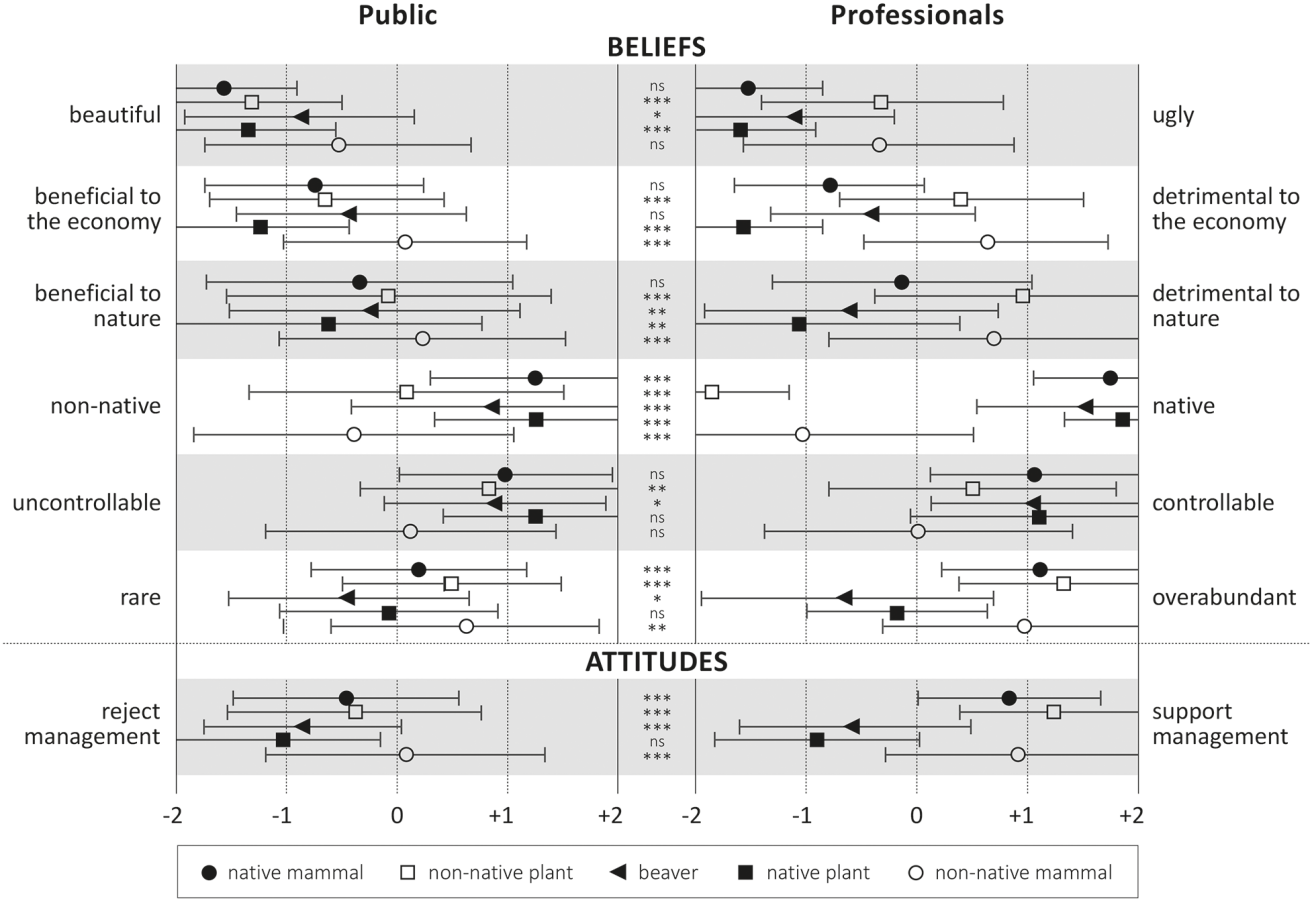
Mean semantic differential scores for six beliefs and for attitude towards management of five species among both public and professionals in Scotland and Ontario. Error bars show standard deviations. Asterisks show significance of difference between public and professionals (*t*-test, two-tailed): ***: *p*<0.001, **: *p*<0.01, *: *p*<0.05. Public sample n = 564, professionals n = 186.

While the public’s and the professionals’ belief scores for the five species were usually on the same side of the spectrum (Fig. 1), differences were often statistically significant. This was, in many cases, due to the tendency of professionals’ beliefs to be more extreme, especially in relation to nativeness and abundance of a species (see e.g., the native mammal, where differences between all other belief scores were n. s.). The divergence in beliefs was particularly pronounced in the case of the non-native plants. On average, professionals perceived rhododendron (Scotland) and Scots pine (Ontario) to be less beautiful, more abundant and detrimental, and less controllable than the public perceived them to be. Not surprisingly, the professionals rated these two species clearly as non-native, whereas the public considered them, on average, as neither native nor non-native (all differences p<0.01, t-test).

There was also a clear divergence in attitudes: for all species except the native plant (Scots pine in Scotland, white pine in Ontario), professionals were significantly (t-test, p<0.001) more in favour of intervention than the public.

### How beliefs inform attitudes: A comparison between professionals and the public

**Figure 2:**
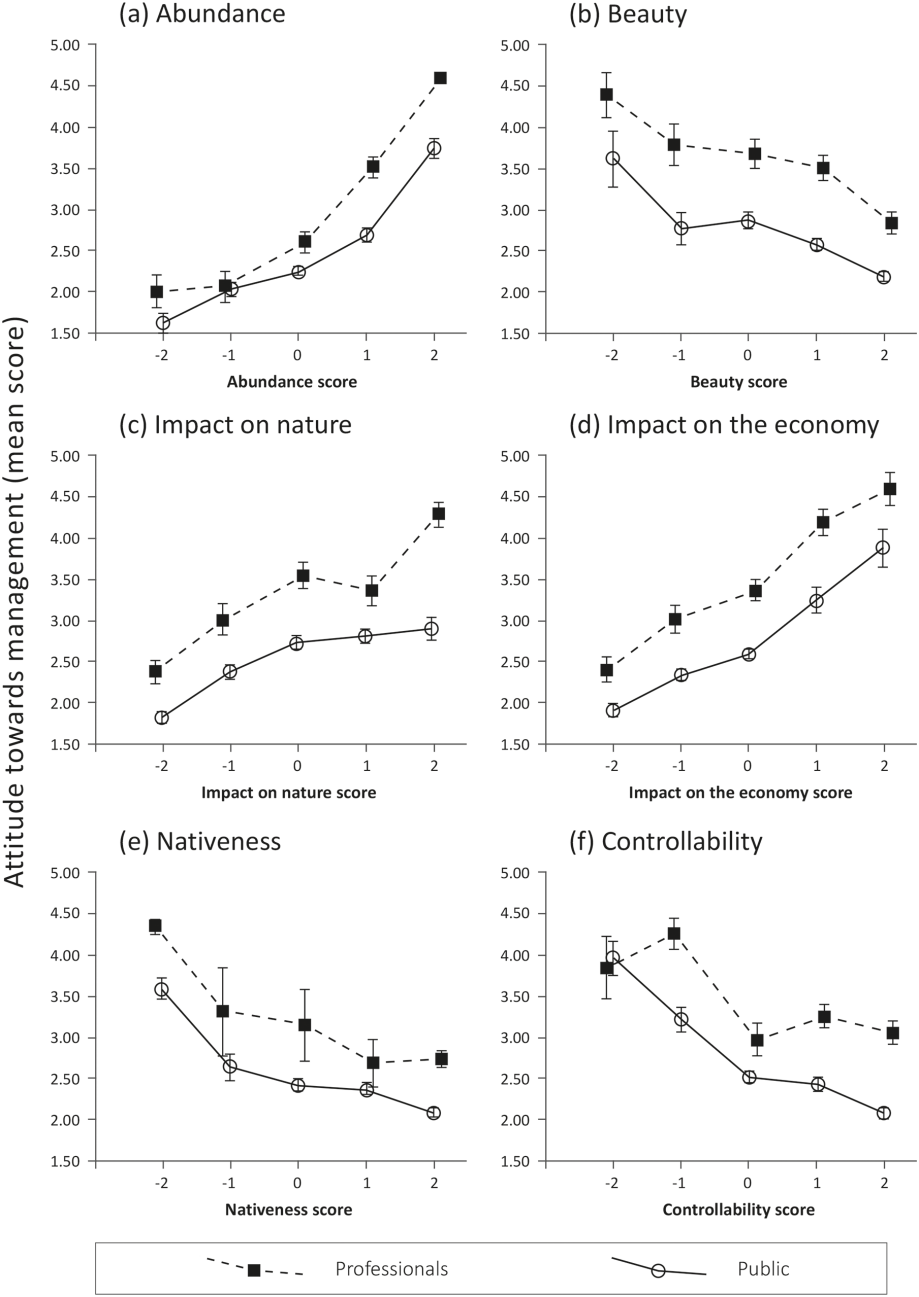
Mean attitude index scores (higher scores = stronger support for management) among both public and professionals in Scotland and Ontario (pooled) towards management of all five species types, in relation to beliefs: (a) abundance, (b) beauty, (c) impact on nature, (d) impact on the economy, (e) nativeness and (f) controllability. Belief variables were coded such that label indicates direction: the higher the score, the more abundant, beautiful, detrimental, native, or controllable. Error bars show 95% confidence interval. Note that lines between points do not denote interpolation, but are added to improve legibility.

All beliefs except controllability were closely related to the attitude scores for both professionals and the public (Fig. 2): when respondents perceived a species to be more abundant (or less beautiful, or less native), they were more likely to strongly support its management. This relationship was also visible for controllability in the public sample – the more people perceived a species to be controllable, the less they felt that management was required – but this was less clear for the professionals. For all other beliefs, these relationships were strikingly similar for the two groups. Overall, professionals tended to have more favourable attitudes towards species management, as indicated by a consistently higher average score in the attitude index.

**Figure 3:**
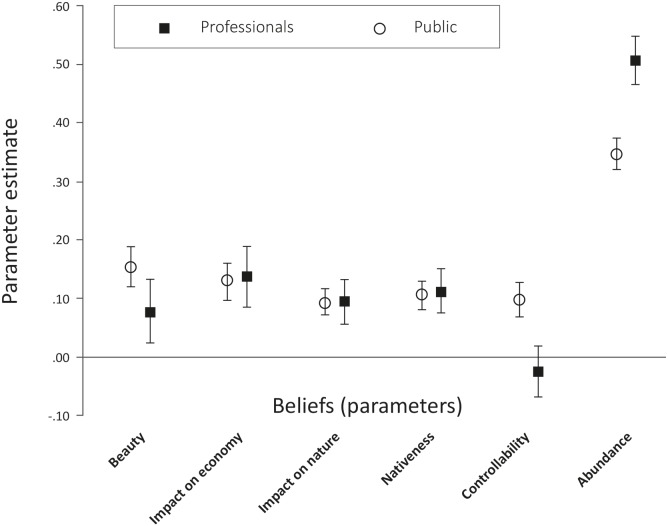
Parameter estimates of fixed effects in a linear mixed model. Type III sums of squares. The dependent variable is attitude towards species management, with higher scores indicating stronger support for management. Belief variables recoded such that parameter estimates are all positive: the higher the score, the more ugly, detrimental, non-native, uncontrollable, abundant the species. The parameter ‘study area’ (Scotland/Ontario) was included in the analysis as a fixed effect (not shown in diagram for clarity). Error bars show 95% confidence interval.

A linear mixed model (Fig. 3, Table 3) confirmed the role of beliefs as explanatory factors: perceived beauty, impact on economy and on nature, nativeness and abundance were all found to contribute significantly (p<0.001) to variance of the dependent variable, i.e., attitudes towards species management. Abundance was by far the most influential factor (F = 1101.71), and even more strongly pronounced in the professionals (parameter estimate = 0.51, i.e., a 0.51 unit increase in the predicted attitude score per one unit increase in the abundance score) than the public (parameter estimate = 0.35). The effects of perceived nativeness, beauty, and impact on the economy and nature had F-values ranging between 50 and 82. Although of similar size to each other, they were thus substantially less powerful explanatory variables than abundance. The only belief that did not add explanatory value was controllability in the professional sample (as already suggested by Fig. 2d). In terms of their direction, significance and relative size, the effects of most beliefs included in the models, except controllability, were thus strikingly similar for the public and professional samples, indicating that people from both groups used beliefs in a similar fashion in their reasoning about species management.

**Table 3:** Fixed effects statistics for linear mixed model.

Parameter	F-value	Significance (*p*)
Intercept	156.11	<0.001
Beauty	50.95	<0.001
Impact on economy	72.52	<0.001
Impact on nature	71.86	<0.001
Nativeness	81.45	<0.001
Controllability	8.35	0.004
Abundance	1101.71	<0.001
Study area (Scotland vs. Canada)	51.76	<0.001
Group	9.17	0.002
Group×Beauty	5.45	0.018
Group×Impact on economy	0.05	0.819
Group×Impact on nature	0.01	0.940
Group×Nativeness	0.09	0.770
Group×Controllability	21.04	<0.001
Group×Abundance	41.82	<0.001
Study area×Group	0.38	0.540

Type III sums of squares. Dependent variable: Attitude towards species management.

The significance of interaction terms (Table 3) adds further detail to the comparison of how beliefs and attitudes relate to one another in the public and professional samples. Interactions of group with ‘controllability’ and ‘beauty’ were significant, with the public more strongly relating these beliefs with their attitudes towards species management, though the effects were relatively small. Because abundance as a main effect was extremely strong, the interaction between abundance and group was also significant, but effectively a rather small ‘correction’ on abundance as a main effect. Importantly, F-values for the interaction between group and the three other beliefs were very small and non-significant. There was therefore no evidence that professionals utilised the beliefs ‘impact on economy’, ‘impact on nature’ and ‘nativeness’ differently than the public when considering the need for management of the focal species.

The variable ‘study area’ took account of variation in attitudes towards management that could not be associated to (beliefs about) species and suggested that on the whole, respondents from Scotland tended to support management action more strongly than those from Ontario. This held true for both professionals and the public as was confirmed by the small and non-significant F-value for ‘study area×group’.

## Discussion

While beliefs and attitudes towards species often differed between the public and professionals (Fig. 1), the way in which these beliefs informed attitudes were very similar across the two groups (Figs. 2, 3; Table 3). In other words, while there were differences in average content knowledge between the two samples, ways of reasoning (as captured in this study) largely concurred. For example, for many species, public and professional views on abundance or nativeness diverged. Yet, relationships between these beliefs and attitudes towards the management of these species were essentially the same among both professionals and the public, except in the case of perceived controllability, as both groups made the same connections between species attributes and the need for management. This means that ways of reasoning about animal and plant species employed by professionals and the public might be much more compatible with each other than commonly thought by invasion biologists, a finding that further expands on previous results that, while the *content* of their thoughts might diverge, ecological professionals and the lay public share the same *structure* of thought about the natural environment in general [4]. Our study thus offers support for a new critical perspective on the ‘deficit model’ of public understanding (see Introduction) that focuses on structure rather than merely on content.

Such commonalities in reasoning could form the starting point for discussions on invasive species management in which both professionals and the public participate on more equal terms. An increase in experts’ trust in non-experts’ ability to form well-founded attitudes towards invasive species management could facilitate the joint development of more widely accepted management options, and thus reduce the current level of public objection to invasive species control [22]. For example, we found that members of the public, in line with widespread thinking in invasion biology, clearly linked the harm caused by a species with the need to manage it. However, our participants’ assessments were made on a case-by-case basis, which might be at odds with a heuristic in invasion biology that contends that all non-native species are potentially harmful and thus require management per se [16] (see [50] for a critical debate): Although there is a widespread understanding that only a fraction of non-native species turn into pests, the uncertainties associated with non-native management have led some invasion biologists to stress the need for general prevention [20] or to argue that all alien species should be considered “guilty until proven innocent” [51]. Our data suggests that the public will appreciate communication on species management that also addresses factors other than non-nativeness and invasiveness – notably a species’ abundance and its effects on nature and the economy (Fig. 3). However, often ‘invasion’ appears to be used as a shorthand that implies a range of other (normative) factors such as harmfulness, which should be made explicit. Some of the apparent differences between public and scientific views, which have been ascribed to “divergent ethical frameworks” [16], could instead be due to such shortcuts in the arguments of invasion biologists that neglect species characteristics which for many people, including professionals in the field, are of importance when assessing the need for species management.

This is not to say that diverging beliefs about species are irrelevant and should be ignored. For example, where perceptions of a species’ abundance differ, these should be addressed, and the causes for conflicting perceptions investigated in more detail. Such conflicts can occur between professionals and the public, as well as between professionals or between members of the public, such as when assessments of the economic value of a species diverge. Yet, a discussion that starts on common ground – namely the recognition that patterns of reasoning are shared, even if content knowledge may differ – has higher chances of success than a polarised debate based on the assumption that the public simply has to be told how to think by professionals in a uni-directional manner (see also [52]).

Our study shows that such patterns of reasoning can also be found implicitly, in the relationships between beliefs and attitudes as expressed in a questionnaire, and not only in the explicit connections made in discussions, as documented in qualitative studies with usually small samples [33], [34]. While our study was built on a simple framework that included only two types of constructs, namely beliefs and attitudes, we have elsewhere investigated more complex mental structures related to biodiversity management, their discursive contexts and a range of decision-making processes [33], [38], [53]. Both beliefs and attitudes can, for example, be conceptualised as part of social representations that develop and are negotiated in individuals’ social context [54]. The degree to which they are actively linked in a concrete situation, such as responding to a questionnaire, can depend on a range of factors, for example, a person’s emotional involvement (i.e., their affective engagement) with the topic [53]. Our previous findings are compatible with the reduced framework adopted in this study, and we thus argue that, where the context is kept in mind, simple frameworks can produce valid insights into more complex relationships.

Our hypotheses proposed that professionals do not consider attractiveness (or beauty) of a species in their thinking about management (see Introduction). However, our results clearly show that they did, albeit to a lesser degree than the public (Figs. 2 and 3). Conversely, we hypothesised that a species’ nativeness and the harm it caused to nature and the economy would play a very strong role in informing professionals’ attitudes towards management, whereas this role would be less pronounced for the general public. Yet, differences between the two groups were not significant (Table 2), again suggesting that conservation biological thinking might be more mainstream among the public than often thought.

Our results also add further evidence to previous studies regarding individual beliefs about invasive species management. Overall, we found that beliefs about nativeness appeared more important for the formation of attitudes than in previous studies [41]. This might be due to the fact that in our analysis, nativeness and abundance were not explicitly separated from the concept of human responsibility in a species’ spread – a notion that earlier qualitative research had identified as important in participants’ talk about invasive species [33]. Future analyses could address the relationship between these closely related and often confounded concepts [52], [55] in more depth. Our results also add evidence to previous studies with members of the general public that identified perceived abundance as the most important factor in informing attitudes towards a species [41]: For professionals, this effect was even stronger than among members of the public, although we can assume that the statistical significance of the difference between the two groups was largely due to the exceptionally high F-value for abundance compared to the F-values for other beliefs (Table 3).

Only in the case of controllability did the relationship between beliefs and attitudes really diverge between the two groups. While there was no significant relation between controllability and support for management among the professionals, members of the public tended to perceive more controllable species as requiring less management than less controllable ones – a result that seems to contradict ideas of risk research in other environmental domains [13]. Nevertheless, this finding is plausible as it suggests that respondents saw a greater cause for concern and need for intervention in those species they considered difficult to control. The absence of a measurable effect in the professional sample might be due to the simultaneous presence of these competing conceptualisations of controllability.

Our strict focus on a particular set of species and two psychological constructs allowed us to identify patterns among human populations through a quantitative approach, but also constrained the scope of the findings. The target species had to be widely familiar because large proportions of ‘don’t know’ responses among both the public and often highly specialised professionals would have rendered the data meaningless. The inclusion of other study areas with possibly more controversial species, such as large carnivores with expanding populations [56], less well-known and less charismatic, or conversely, culturally or economically significant non-native plant species [23], could have led to even larger differences between professionals and the public in beliefs or attitudes. However, there is no reason to suspect that the *relationships* between beliefs and attitudes would be affected by specific species, because similar relationships have also been found for other species and other European countries [41]. While our set of species would ideally be larger for the conclusions to be more readily transferable (but for pragmatic reasons, the length of a questionnaire is obviously limited), our study goes substantially beyond previous research that often addressed only one or two species [23], [25]. This notwithstanding, qualitative research will always be required to provide more in-depth insights into cultural or symbolic meanings of species.

In summary, we conclude that in countries such as Great Britain and Canada, differences between public and professional views on invasive species management are unlikely to result from fundamental differences in reasoning (as captured in this study). Instead, such divergences may either be caused by diverging beliefs about species, or by procedural aspects related to communication, decision-making or species management. In both cases, we suggest that disputes about invasive species management may be reduced by increased transparency and a more differentiated debate that makes use of shared understandings of relationships between species characteristics and the need for intervention.

## Supporting Information

Questionnaire S1 **Questionnaire for public sample in Scotland.**
(PDF)

## References

[1] Wynne BE (1996) May the sheep safely graze? A reflexive view of the expert-lay knowledge divide. In: Lash S, Szerszynksi B, Wynne B, editors. Risk, environment and modernity: towards a new ecology. London: Sage. 44–82.

[2] Rauschmayer F, van den Hove S, Koetz T (2008) Participation in EU biodiversity governance: How far beyond rhetoric? Environment and Planning C 27: 42–58.

[3] Hunziker M, Felber P, Gehring K, Buchecker M, Bauer N, et al (2008) Evaluation of landscape change by different social groups. Mountain Research and Development 28: 140–147.

[4] Buijs AE, Elands BHM (2013) Does expertise matter? An in-depth understanding of people’s structure of thoughts on nature and its management implications. Biological Conservation 168: 184–191.

[5] Dallimer M, Irvine KN, Skinner AMJ, Davies ZG, Rouquette JR, et al (2012) Biodiversity and the feel-good factor: Understanding associations between self-reported human well-being and species richness. BioScience 62: 47–55.

[6] Besley JC, Nisbet M (2011) How scientists view the public, the media and the political process. Public Understanding of Science 20: 1–16. 10.1177/0963662511418743 23885050

[7] Gieryn TF (1983) Boundary-work and the demarcation of science from non-science: strains and interests in professional ideologies of scientists. American Sociological Review 48: 781–795.

[8] Trumbull DJ, Bonney R, Bascom D, Cabral A (2000) Thinking scientifically during participation in a citizen-science project. Science Education 84: 265–275.

[9] Bell M, Sheail J (2005) Experts, publics and the environment in the UK: twentieth-century translations. Journal of Historical Geography 31: 496–512.

[10] Giddens A (1994) Living in a post-traditional society. In: Beck U, Giddens A, Lash S, editors. Reflexive modernization: politics, tradition and aesthetics in the modern social order. Cambridge: Polity Press. 56–109.

[11] Roux D J, Rogers KH, Biggs HC, Ashton PJ, Sergeant A (2006) Bridging the science-management divide: Moving from unidirectional knowledge transfer to knowledge interfacing and sharing. Ecology and Society 11: 4.

[12] Larson B (2011) Metaphors for Environmental Sustainability: Redefining our Relationship with Nature. New Haven: Yale University Press.

[13] McDaniels TL, Axelrod LJ, Cavanagh NS, Slovic P (1997) Perception of ecological risk to water environments. Risk Analysis 17: 341–352. 9232017 10.1111/j.1539-6924.1997.tb00872.x

[14] Tveit MS (2009) Indicators of visual scale as predictors of landscape preference: a comparison between groups. Journal of Environmental Management 90: 2882–2888. 18951696 10.1016/j.jenvman.2007.12.021

[15] Dandy N, Van der Wal R (2011) Shared appreciation of woodland landscapes by land management professionals and lay people: An exploration through field-based interactive photo-elicitation. Landscape and Urban Planning 102: 43–53.

[16] Simberloff D, Martin JL, Genovesi P, Maris V, Wardle DA, et al (2013) Impacts of biological invasions: what’s what and the way forward. Trends in Ecology and Evolution 28: 58–66. 22889499 10.1016/j.tree.2012.07.013

[17] Larson BMH (2007) An alien approach to invasive species: Objectivity and society in invasion biology. Biological Invasions 9: 947–956.

[18] Young AM, Larson BMH (2011) Clarifying debates in invasion biology: A survey of invasion biologists. Environmental Research 111: 893–898. 21757195 10.1016/j.envres.2011.06.006

[19] Humair F, Edwards PJ, Siegrist M, Kueffer C (2014) Understanding misunderstandings in invasion science: why experts don’t agree on common concepts and risk assessments. NeoBiota 20: 1–30.

[20] Hulme PE (2006) Beyond control: Wider implications for the management of biological invasions. Journal of Applied Ecology 43: 835–847.

[21] DEFRA (Department for Environment, Food and Rural Affairs) (2008) The Invasive Non-Native Species Framework Strategy for Great Britain. DEFRA, London.

[22] Perry D, Perry G (2008) Improving interactions between animal rights groups and conservation biologists. Conservation Biology 22: 27–35. 18254850 10.1111/j.1523-1739.2007.00845.x

[23] Marshall NA, Friedel M, Van Klinken RD, Grice AC (2011) Considering the social dimension of invasive species: the case of buffel grass. Environmental Science and Policy 14: 327–338.

[24] Bremner A, Park K (2007) Public attitudes to the management of invasive non-native species in Scotland. Biological Conservation 139: 306–314.

[25] Fischer A, Van der Wal R (2007) Invasive plant suppresses charismatic seabird – the construction of attitudes toward biodiversity management options. Biological Conservation 135: 256–267.

[26] Somaweera R, Somaweera N, Shine R (2010) Frogs under friendly fire: How accurate can the general public recognize invasive species? Biological Conservation 143: 1477–1484.

[27] Sharp RL, Larson LR, Green GT (2011) Factors influencing public preferences for invasive alien species management. Biological Conservation 144: 2097–2104.

[28] Schüttler E, Rozzi R, Jax K (2011) Towards a societal discourse on invasive species management: A case study of public perceptions of mink and beavers in Cape Horn. Journal for Nature Conservation 19: 175–184.

[29] Verbrugge LNH, Van den Born RJG, Lenders HJR (2013) Exploring public perceptions of non-native species from a visions of nature perspective. Environmental Management 52: 1562–1573. 24071727 10.1007/s00267-013-0170-1

[30] Andreu J, Vilá M, Hulme P (2009) An assessment of stakeholder perceptions and management of noxious alien plants in Spain. Environmental Management 43: 1244–1255. 19214625 10.1007/s00267-009-9280-1

[31] Bardsley DK, Edwards-Jones G (2007) Invasive species policy and climate change: Social perceptions of environmental change in the Mediterranean. Environmental Science and Policy 10: 230–242.

[32] García-Llorente M, Martín-López B, González JA, Alcorlo P, Montes C (2008) Social perceptions of the impacts and benefits of invasive alien species: Implications for management. Biological Conservation 141: 2969–2983.

[33] Selge S, Fischer A, Van der Wal R (2011) Public and professional views on invasive non-native species - a qualitative social scientific investigation. Biological Conservation 144: 3089–3097.

[34] Sadler TD, Zeidler DL (2005) The significance of content knowledge for informal reasoning regarding socioscientific issues: Applying genetics knowledge to genetic engineering issues. Science Education 89: 71–93.

[35] Jordan RC, Gray SA, Howe DV, Brooks WR, Ehrenfeld JG (2011) Knowledge gain and behavioural change in citizen-science programs. Conservation Biology 25: 1148–1154. 21967292 10.1111/j.1523-1739.2011.01745.x

[36] Ajzen I (1988) Attitudes, Personality, and Behavior. Open University Press, Milton Keynes.

[37] Milfont TL, Duckitt J (2010) The environmental attitudes inventory: A valid and reliable measure to assess the structure of environmental attitudes. Journal of Environmental Psychology 30: 80–94.

[38] Fischer A, Langers F, Bednar-Friedl B, Geamana N, Skogen K (2011) Mental representations of animal and plant species in their social contexts: Results from a survey across Europe. Journal of Environmental Psychology 31: 118–128.

[39] Montgomery CA (2002) Ranking the benefits of biodiversity: An exploration of relative values. Journal of Environmental Management 65: 313–326. 12357662 10.1006/jema.2002.0553

[40] Gobster PH (2011) Factors affecting people’s responses to invasive species management. In: Rotherham ID, Lambert RA, editors. Invasive and introduced plants and animals – human perceptions, attitudes and approaches to management. London: Earthscan. 249–263.

[41] Fischer A, Bednar-Friedl B, Langers F, Geamana N, Skogen K, et al (2011) Universal criteria for species conservation priorities? Findings from a survey of public views across Europe. Biological Conservation 144: 998–1007.

[42] Buijs AE, Arts BJM, Elands BHM, Langkeek J (2011) Beyond environmental frames: The social representation and cultural resonance of nature in conflicts over a Dutch woodland. Geoforum 42: 329–341.

[43] Peel MC, Finlayson BL, McMahon TA (2007) Updated world map of the Köppen-Geiger climate classification. Hydrology and Earth System Sciences 11: 1633–1644.

[44] Colautti RI, MacIsaac HJ (2004) A neutral terminology to define ‘invasive’ species. Diversity and Distribution 10: 135–141.

[45] Jeschke JM, Strayer DL (2005) Invasion success of vertebrates in Europe and North America. Proceedings of the National Academy of Sciences 102: 7198–7202. 10.1073/pnas.0501271102 PMC1129111 15849267

[46] Lodge DM, Williams S, MacIsaac H, Hayes KR, Leung B, et al (2006) Biological invasions: Recommendations for U.S. policy and management. Ecological Applications 16: 2035–2054. 17205888 10.1890/1051-0761(2006)016[2035:birfup]2.0.co;2

[47] Naughton D (2012) The Natural History of Canadian Mammals. Toronto: Canadian Museum of Nature and University of Toronto Press.

[48] Osgood CE (1952) The nature and the measurement of meaning. Psychological Bulletin 49: 197–237. 14930159 10.1037/h0055737

[49] Fulton DC, Manfredo MJ, Lipscomb J (1996) Wildlife Value Orientations: A Conceptual and Measurement Approach. Human Dimensions of Wildlife 1: 24–47.

[50] Davis MA, Chew MK, Hobbs RJ, Lugo AE, Ewel JJ, et al (2011) Don’t judge species on their origins. Nature 474: 153–154. 21654782 10.1038/474153a

[51] Simberloff D (2007) Given the stakes, our *modus operandi* in dealing with invasive species should be “guilty until proven innocent”. Conservation Magazine 8: 18–19.

[52] Davis MA (2009) Invasion biology. Oxford: Oxford University Press.

[53] Fischer A, Glenk K (2011) One model fits all? On the moderating role of emotional engagement and confusion in the elicitation of preferences for climate change adaptation policies. Ecological Economics 70: 1178–1188.

[54] Buijs AE, Hovardas T, Castro P, Devine-Wright P, Figari H, et al (2012) Understanding people’s ideas on natural resource management: research on social representations of nature and the environment. Society and Natural Resources 25: 1167–1181.

[55] Warren CR (2007) Perspectives on the ‘alien’ versus ‘native’ species debate: A critique of concepts, language and practice. Progress in Human Geography 31: 427–446.

[56] Skogen K, Thrane C (2008) Wolves in context. Using survey data to situate attitudes within a wider cultural framework. Society and Natural Resources 21: 17–33.

