# Supplementary material for: The Public and Professionals Reason Similarly about the Management of Non-Native Invasive Species: A Quantitative Investigation of the Relationship between Beliefs and Attitudes
Source: PLoS One. 2014 Aug 29;9(8):e105495. doi: 10.1371/journal.pone.0105495 (PMC4149425; doi:10.1371/journal.pone.0105495)
Supplement: Questionnaire S1 — Questionnaire for public sample in Scotland. (PDF) [file pone.0105495.s001.pdf]

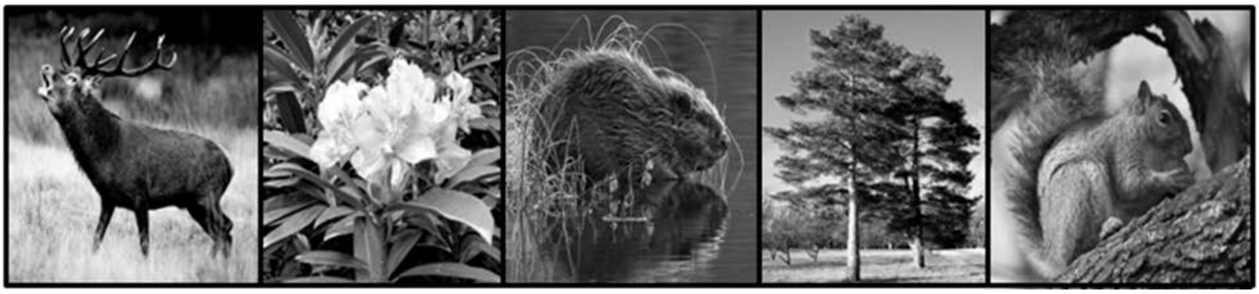

Your views on animal and plant species in Scotland

Aberdeen, June 2010

THE MACAULAY LAND USE RESEARCH INSTITUTE

1495 UNIVERSITY OF ABERDEEN

Dear participant,

Thank you very much for your interest in this questionnaire!

What is the purpose of this research?

Recently, there has been a lot of discussion about changes in our natural environment, for example, changes in animal and plant populations in Scotland. In some cases, you might feel that something should be done. However, there is a wide range of options that policymakers and landmanagers can choose to manage animal and plant species. What is your view?

This questionnaire aims to find out more about what is important to people when they make judgments about species and their management. This knowledge is essential to improve communication between the public and policymakers.

What are we asking you to do?

We will ask you questions about your views on:

1) Environmental changes in Scotland
2) Several plant and animal species
3) How you see the relationship between humans and nature.

Answering the questionnaire will take about 20 minutes. Please follow the instructions carefully:

1) The questions should be filled in by one person only, without consulting others.
2) Unless otherwise mentioned, you can only choose one answer.
3) Don’t skip any questions! It is important for us that you answer every question.
4) In case you feel the question is <u>not applicable</u> to the species or you <u>don’t know</u> what to answer, please make use of the “don’t know” option.
5) Don’t think too long about the questions. In most cases, your first impression will be the best.

There are no right or wrong responses! We are only interested in your personal opinion.

What will happen with the information you provide?

Of course, the information you give will be kept absolutely confidential and analysed carefully. A summary of the results will be sent to Scottish Natural Heritage.

Thank you for your help! We are very grateful for your response.

Sebastian Selge (for questions and concerns please call: 01224-395-308)

Below are statements regarding current environmental changes in Scotland. In your view, do policymakers in Scotland pay enough attention to the following topics?

	not enough		enough		too much
species extinctions	O	O	O	O	O
reforestation	O	O	O	O	O
pollution	O	O	O	O	O
people's awareness of nature	O	O	O	O	O
global warming	O	O	O	O	O
conservation projects	O	O	O	O	O
non-native species	O	O	O	O	O
organic farming	O	O	O	O	O

In case you feel we missed out an environmental topic that is very important to you, please write it down here:

____

____

____

In the following section you will see questions that look like this:

**I like red deer**	O	X	O	O	O	**I don't like red deer**

If you feel you like red deer very much, please make a cross on the left hand end of the scale next to "I like red deer". However, if you don't like red deer at all cross the circle on the other side next to "I don't like red deer". If your views fall somewhere in between, please choose the appropriate circle between the extremes. If you feel the question **cannot** be answered or if you do not know what to answer, please use the "**don't know N/A**"-option! All questions refer to Scotland.

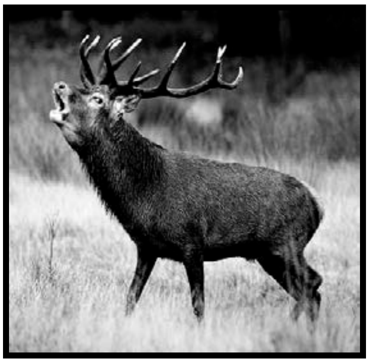

Red deer

I am familiar with red deer	O	O	O	O	O	I am unfamiliar with red deer
I like red deer	O	O	O	O	O	I don't like red deer

In your view, how would you describe red deer with respect to the following attributes?

							don't know N/A
ugly	O	O	O	O	O	beautiful	O
detrimental to the economy	O	O	O	O	O	beneficial to the economy	O
beneficial to nature	O	O	O	O	O	detrimental to nature	O
non-native	O	O	O	O	O	native	O
accidentally introduced	O	O	O	O	O	intentionally introduced	O
uncontrollable	O	O	O	O	O	controllable	O
rare	O	O	O	O	O	overabundant	O
introduced by humans	O	O	O	O	O	natural range expansion	O
<u>not</u> a severe problem	O	O	O	O	O	a severe problem	
need to reduce numbers	O	O	O	O	O	<u>no</u> need to reduce numbers	
killing red deer is <u>not</u> ok	O	O	O	O	O	killing red deer is ok	
has same rights as humans	O	O	O	O	O	has <u>not</u> same rights as humans	

When deciding if the red deer requires management, how important are the following categories to **you**?

	not at all important	un-important	neither nor	important	very important
beautiful/ugly	O	O	O	O	O
beneficial/detrimental to economy	O	O	O	O	O
beneficial/detrimental to nature	O	O	O	O	O
native/non-native	O	O	O	O	O
intentionally/accidentally introduced	O	O	O	O	O
controllable/uncontrollable	O	O	O	O	O
rare/overabundant	O	O	O	O	O
human-caused/natural	O	O	O	O	O

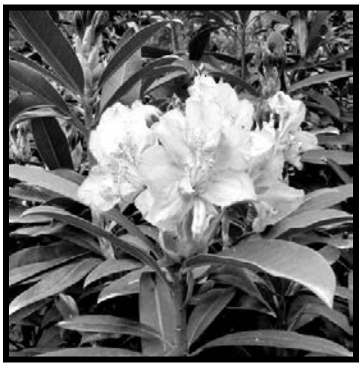

Rhododendron ponticum

I am familiar with rhododendron	O	O	O	O	O	I am unfamiliar with rhododendron
I like rhododendron	O	O	O	O	O	I don’t like rhododendron

In your view, how would you describe rhododendron with respect to the following attributes?

							don’t know N/A
ugly	O	O	O	O	O	beautiful	O
detrimental to the economy	O	O	O	O	O	beneficial to the economy	O
beneficial to nature	O	O	O	O	O	detrimental to nature	O
non-native	O	O	O	O	O	native	O
accidentally introduced	O	O	O	O	O	intentionally introduced	O
uncontrollable	O	O	O	O	O	controllable	O
rare	O	O	O	O	O	overabundant	O
introduced by humans	O	O	O	O	O	natural range expansion	O
not a severe problem	O	O	O	O	O	a severe problem	
need to reduce numbers	O	O	O	O	O	no need to reduce numbers	
killing rhododendron is not	O	O	O	O	O	killing rhododendron is ok	
has same rights as humans	O	O	O	O	O	has not same rights as humans	

When deciding if rhododendron require management, how important are the following categories to **you**?

	not at all important	un-important	neither nor	important	very important
beautiful/ugly	O	O	O	O	O
beneficial/detrimental to economy	O	O	O	O	O
beneficial/detrimental to nature	O	O	O	O	O
native/non-native	O	O	O	O	O
intentionally/accidentally introduced	O	O	O	O	O
controllable/uncontrollable	O	O	O	O	O
rare/overabundant	O	O	O	O	O
human-caused/natural	O	O	O	O	O

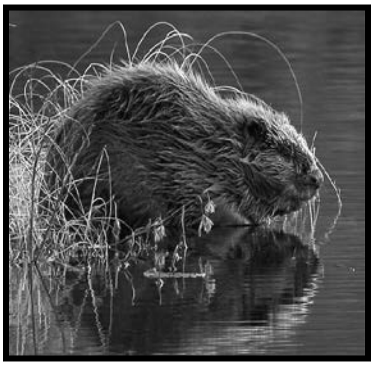

Beaver

I am familiar with the beaver	O	O	O	O	O	I am unfamiliar with the beaver
I like beavers	O	O	O	O	O	I don't like beavers

In your view, how would you describe the beaver with respect to the following attributes?

							don't know N/A
ugly	O	O	O	O	O	beautiful	O
detrimental to the economy	O	O	O	O	O	beneficial to the economy	O
beneficial to nature	O	O	O	O	O	detrimental to nature	O
non-native	O	O	O	O	O	native	O
accidentally introduced	O	O	O	O	O	intentionally introduced	O
uncontrollable	O	O	O	O	O	controllable	O
rare	O	O	O	O	O	overabundant	O
introduced by humans	O	O	O	O	O	natural range expansion	O
not a severe problem	O	O	O	O	O	a severe problem	
need to reduce numbers	O	O	O	O	O	no need to reduce numbers	
killing beavers is not ok	O	O	O	O	O	killing beavers is ok	
has same rights as humans	O	O	O	O	O	has not same rights as humans	

When deciding if beaver require management, how important are the following categories to **you**?

	not at all important	un-important	neither nor	important	very important
beautiful/ugly	O	O	O	O	O
beneficial/detrimental to economy	O	O	O	O	O
beneficial/detrimental to nature	O	O	O	O	O
native/non-native	O	O	O	O	O
intentionally/accidentally introduced	O	O	O	O	O
controllable/uncontrollable	O	O	O	O	O
rare/overabundant	O	O	O	O	O
human-caused/natural	O	O	O	O	O

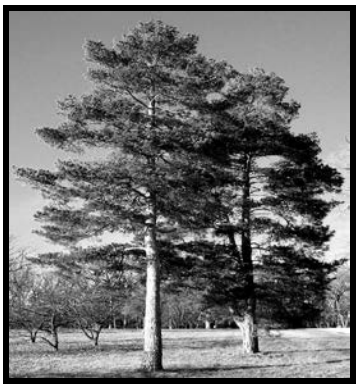

Scots pine

I am familiar with Scots pine	O	O	O	O	O	I am unfamiliar with Scots pine
I like Scots pine	O	O	O	O	O	I don't like Scots pine

In your view, how would you describe Scots pine with respect to the following attributes?

							don't know N/A
ugly	O	O	O	O	O	beautiful	O
detrimental to the economy	O	O	O	O	O	beneficial to the economy	O
beneficial to nature	O	O	O	O	O	detrimental to nature	O
non-native	O	O	O	O	O	native	O
accidentally introduced	O	O	O	O	O	intentionally introduced	O
uncontrollable	O	O	O	O	O	controllable	O
rare	O	O	O	O	O	overabundant	O
introduced by humans	O	O	O	O	O	natural range expansion	O
<u>not</u> a severe problem	O	O	O	O	O	a severe problem	
need to reduce numbers	O	O	O	O	O	<u>no</u> need to reduce numbers	
killing Scots pine is <u>not</u> ok	O	O	O	O	O	killing Scots pine is ok	
has same rights as humans	O	O	O	O	O	has <u>not</u> same rights as humans	

When deciding if Scots pine requires management, how important are the following categories to you?

	not at all important	un-important	neither nor	important	very important
beautiful/ugly	O	O	O	O	O
beneficial/detrimental to economy	O	O	O	O	O
beneficial/detrimental to nature	O	O	O	O	O
native/non-native	O	O	O	O	O
intentionally/accidentally introduced	O	O	O	O	O
controllable/uncontrollable	O	O	O	O	O
rare/overabundant	O	O	O	O	O
human-caused/natural	O	O	O	O	O

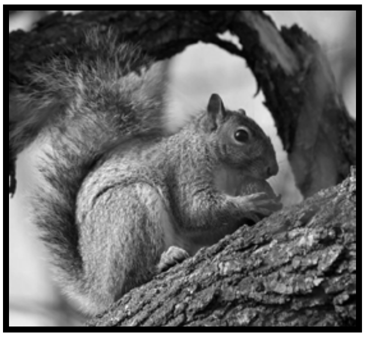

Grey squirrel

I am familiar with the grey squirrel	O	O	O	O	O	I am unfamiliar with the grey squirrel
I like the grey squirrel	O	O	O	O	O	I don’t like the grey squirrel

In your view, how would you describe the grey squirrel with respect to the following attributes?

							don’t know N/A
ugly	O	O	O	O	O	beautiful	O
detrimental to the economy	O	O	O	O	O	beneficial to the economy	O
beneficial to nature	O	O	O	O	O	detrimental to nature	O
non-native	O	O	O	O	O	native	O
accidentally introduced	O	O	O	O	O	intentionally introduced	O
uncontrollable	O	O	O	O	O	controllable	O
rare	O	O	O	O	O	overabundant	O
introduced by humans	O	O	O	O	O	natural range expansion	O
<u>not</u> a severe problem	O	O	O	O	O	a severe problem	
need to reduce numbers	O	O	O	O	O	<u>no</u> need to reduce numbers	
killing grey squirrles is <u>not</u>	O	O	O	O	O	killing grey squirrles is ok	
has same rights as humans	O	O	O	O	O	has <u>not</u> same rights as humans	

When deciding if grey squirrels require management, how important are the following categories to **you**?

	not at all important	un-important	neither nor	important	very important
beautiful/ugly	O	O	O	O	O
beneficial/detrimental to economy	O	O	O	O	O
beneficial/detrimental to nature	O	O	O	O	O
native/non-native	O	O	O	O	O
intentionally/accidentally introduced	O	O	O	O	O
controllable/uncontrollable	O	O	O	O	O
rare/overabundant	O	O	O	O	O
human-caused/natural	O	O	O	O	O

Now, we would like to ask you about your views on nature and life in general. Again, this is about your personal opinion and there are no right or wrong responses.

	strongly disagree	disagree	neutral	agree	strongly agree
I believe if humans mistreat nature, they will suffer environmental catastrophes	O	O	O	O	O
Only if we treat nature with respect will humankind be able to survive	O	O	O	O	O
I care about animals as I do about other people	O	O	O	O	O
I think humankind cannot expect a future worth living if we are abusing nature	O	O	O	O	O
I don't think that respect for nature will save us from any environmental catastrophe	O	O	O	O	O
I feel a strong emotional bond with nature	O	O	O	O	O
I think humans don't deserve benefits from nature, if they mistreat it	O	O	O	O	O
Animals should have rights similar to the rights of humans	O	O	O	O	O
Environmental catastrophes are even worse when humans are responsible for them	O	O	O	O	O
I view all living things as part of one big family	O	O	O	O	O
I think basically the world is a just place	O	O	O	O	O
I believe that, by and large, people get what they deserve	O	O	O	O	O
I am confident that justice always prevails over injustice	O	O	O	O	O
I am confident that in the long run, people will be compensated for injustices	O	O	O	O	O
I firmly believe that injustices in all areas of life (e.g. professional, family, politics) are the exception rather than the rule	O	O	O	O	O
I think people try to be fair when making important decisions	O	O	O	O	O

Now follow a few questions about you. It is important to us that you answer these questions as well so that we can understand how different groups in society view these issues.

How old are you? ________years I am o female o male

In which country did you grow up? ________________________

I get most of my information regarding nature, animals and plants from: (you may tick more than one option!)	O	Newspapers
	O	Television/Radio
	O	Own observations
	O	School, university
	O	Leaflets/magazines from governmental organisations
	O	Leaflets/magazines from non-governmental organisations
	O	Internet
	O	Conversations with friends or family
	O	Scientific journals
	O	Others, namely:

What is your highest educational attainment?	O	Primary school
	O	Secondary school
	O	Sixth form
	O	HND
	O	Degree (e.g. BSc/Ba)
	O	Higher degree (Ms/PhD)

What describes your daily occupation best?	O	Employed full-time
	O	Employed part-time
	O	Self-employed
	O	Unemployed
	O	Retired, pensioner
	O	Homemaker
	O	Studying/vocational training
	O	Others, namely:

How would you describe the area you live in?	O	City
	O	Small town
	O	Rural area

How would you describe yourself?

I would consider myself...	strongly disagree	disagree	neutral	agree	strongly agree
a farmer	O	O	O	O	O
a conservationist	O	O	O	O	O
a nature-loving person	O	O	O	O	O
a birdwatcher	O	O	O	O	O
a outdoor sport enthusiast	O	O	O	O	O
a gardener	O	O	O	O	O
others:________________	O	O	O	O	O

If you have any remarks or comments please write them down here:

____

____

____

____

Thank you very much for your participation!

Please return the completed questionnaire by 23 June 2010 using the FREEPOST envelope provided.

Sebastian Selge
Socio-Economic Research Group
Macaulay Land Use Research Institute
Craigiebuckler
Aberdeen AB15 8QH

Please return the completed questionnaire by 23 June 2010 using the FREEPOST envelope provided.

Many thanks!
